# A Multi-Branch Convolutional Neural Network with Squeeze-and-Excitation Attention Blocks for EEG-Based Motor Imagery Signals Classification

**DOI:** 10.3390/diagnostics12040995

**Published:** 2022-04-15

**Authors:** Ghadir Ali Altuwaijri, Ghulam Muhammad, Hamdi Altaheri, Mansour Alsulaiman

**Affiliations:** 1 Department of Computer Engineering, College of Computer and Information Sciences (CCIS), King Saud University, Riyadh 11543, Saudi Arabia; 438203980@student.ksu.edu.sa (G.A.A.); haltaheri@ksu.edu.sa (H.A.); msuliman@ksu.edu.sa (M.A.); 2 Centre of Smart Robotics Research (CS2R), King Saud University, Riyadh 11543, Saudi Arabia

**Keywords:** attention network, brain-computer interfaces, convolutional neural networks, deep learning, electroencephalography, motor imagery

## Abstract

Electroencephalography-based motor imagery (EEG-MI) classification is a critical component of the brain-computer interface (BCI), which enables people with physical limitations to communicate with the outside world via assistive technology. Regrettably, EEG decoding is challenging because of the complexity, dynamic nature, and low signal-to-noise ratio of the EEG signal. Developing an end-to-end architecture capable of correctly extracting EEG data’s high-level features remains a difficulty. This study introduces a new model for decoding MI known as a Multi-Branch EEGNet with squeeze-and-excitation blocks (MBEEGSE). By clearly specifying channel interdependencies, a multi-branch CNN model with attention blocks is employed to adaptively change channel-wise feature responses. When compared to existing state-of-the-art EEG motor imagery classification models, the suggested model achieves good accuracy (82.87%) with reduced parameters in the BCI-IV2a motor imagery dataset and (96.15%) in the high gamma dataset.

## 1. Introduction

A brain-computer interface (BCI) is a computer-based system that collects, examines, and converts brain signals into instructions that are communicated to an output device to perform a requested response. Brain impulses can now be used to operate devices, owing to advancements in this field [1]. Electroencephalography (EEG) is the most utilized brain signal because it is measured from the scalp (non-invasive), is low cost, and has a high time resolution [2]. Due to the non-stationary nature of EEG signals, their increased susceptibility to artifacts, and their frequent exposure to external noise, processing them is a tough task. Additionally, the subject’s posture and attitude can affect the EEG readings [3].

The electrical activity of the brain recorded from the scalp is the EEG signal, which is made up of several underlying base frequencies. Specific emotional, cognitive, or attentional states are indicated by these frequencies. A frequency range of 0–35 Hz was used in most of the research [4].

This study concentrated on EEG signals derived from motor imagery (MI), the process of imagining limb movement. When a subject imagines moving the right or left hand, or both, or the right or left foot, or any of the five fingers, or the tongue, or any other limb in the human body, MI data are generated. Researchers demonstrated in the early 2000s that the most effective strategy for detecting EEG-based MI was to employ common spatial patterns (CSP). The purpose of the CSP algorithm is to identify a set of linear transformations, frequently referred to as spatial filters, that optimize distance over several classes. The motor imagery of the right hand, left hand, and feet that were recorded during an MI-EEG task are classified into these classes. The data representation is constructed using the relative energy of the filtered channels after the spatial filters have been estimated. For high accuracy, these multi-dimensional EEG data representation can be simply entered into a linear classifier, such as a support vector machine (SVM) [5].

MI-designated EEG as a growing area of interest in the field of BCI is associated with not only enormous potential but also vital applications (e.g., gaming [6], robotics [7,8], and therapeutic applications [9,10]). There are, however, significant limitations in terms of data collecting and categorization techniques. The objective of this research is to develop an end-to-end classification model based on deep learning that is capable of reliably categorizing MI-EEG-based signals with high kappa values, which is a measure of how much agreement can be anticipated by chance. Despite deep learning’s growing popularity in a variety of fields, it has yet to produce satisfying results when used to classify EEG signal-based motor imagery. The high dimensionality of EEG data (multichannel and sampling rate), the presence of artifacts (such as motion), noise, and channel correlation make the design of an optimum EEG classification model using deep learning (DL) difficult.

According to preliminary observations, the main difficulty with EEG MI classification is that it is a more subject-specific task. This means that each person has unique traits that aid the system in correctly classifying the MI movement. This issue can be addressed through the use of multi-scale, multi-branch, or parallel architectures, which increase the model’s generality. However, this type of model is typically computationally expensive, requiring a larger number of parameters and a longer training period. As a result, we present in this paper a DL-based EEG MI classification model that is lightweight and capable of dealing with subject-specific tasks using fixed hyperparameters, making it more suitable for use in real-world applications. The following are the primary contributions of the paper:

- Build an end-to-end multi-branch EEG MI classification model based on DL that can solve the subject-specific problem.
- Develop a lightweight multi-branch attention model that can accurately classify EEG MI signals with a small number of parameters.
- Create a robust general model with fixed hyperparameters.
- Using multiple datasets, test the usefulness and robustness of the proposed model against data fluctuations.

In Section 2, we provide a summary of related research publications on MI-EEG classification algorithms. Section 3 presents the proposed model, multi-branch EEGNet with squeeze-and-excitation block (MBEEGSE), while Section 4 and Section 5 contain a discussion of the experimental data and results, and a conclusion, respectively.

## 2. Related Works

With just one processing block, deep learning can complete the whole feature extraction, selection, and classification pipeline. Convolutional neural networks (CNNs) [11,12,13,14] are the most frequently used architecture in MI EEG processing, but other architectures like recurrent neural networks (RNNs) [12,15], deep belief networks (DBNs) [12], and stacked autoencoders (SAEs) [13] have been utilized as well. Due to the nonlinear and non-stationary nature of EEG MI signals, CNN has an advantage over other deep learning techniques. They possess temporal and spatial features as a result of the time spent visualizing the movement and the simultaneous acquisition of data from several electrodes, each electrode has different locations that contain the spatial information. For that, CNN provides several advantages for analyzing MI EEG data, including high accuracy on large datasets, the ability to exploit the hierarchical nature of particular signals, and the ability to learn both temporal and spatial information concurrently.

Numerous studies used data preparation procedures before feeding information into a CNN. ConvNet [16], which uses convolutional layers to extract temporal and spatial information and was inspired by the filter-bank CSP (FBCSP) [17], was the first interesting technique that used raw EEG data. Two comparable MI topologies were introduced in [18]: the ShallowConvNet, which is a shallow convolutional network with two convolutional layers and classification layers, and the DeepConvNet, which is a deep design with additional aggregating layers. The EEGNet was proposed in [19] as a compact version of previous approaches. It is based on depth-wise convolution and separable convolution, which minimizes the network’s parameter count. Following that, similar structures were proposed, one of which was published by Riyad et al. in [20]. The first half of the model is identical to EEGNet, with the second half containing an inception block. To improve the performance of EEGNet, the researchers applied temporal convolutional networks (TCNs) in [21]. All of these architectures address the shortcomings in EEGNet, such as its shallow and compact design, which restricts network capacity and, in most situations, leads to overfitting. Due to a degradation issue, performance remains low even with a deeper network. As a result, it is recommended to utilize a multibranch model that incorporates attributes from different branches.

In [22], Amin et al. combined multilayer CNNs with two separate feature fusion techniques: a multilayer perceptron (MLP) and autoencoders to produce a new approach to EEG signal classification. The authors examined different levels of CNNs to extract the most significant features, and then combined them before classification to improve the accuracy of EEG-based MI. Their models were trained on the high gamma dataset (HGD) to avoid overfitting. In [23], the same researcher presented an attention-based inception model that contains two attention blocks. Each attention block comprises three parallel convolutions with varying filter sizes, followed by an attention vector that fuses all of the features collected from the convolution process. As demonstrated in [24], a 3D CNN is used in EEG-based MI because it improves classification in image/video processing applications. In [24], Zhao et al. proposed a multi 3D CNN for preserving spatial and temporal properties. They depicted 3D EEG as a sequence of 2D arrays based on the electrode placements, then extended the array to a 3D array using the temporal information from the EEG.

We noticed that no previous research had been done on raw MI-EEG signals as input for 2D CNNs with a multi-branch. In [24,25], the authors used a multi-branch architecture with 3D CNN, with a 3D EEG signal as the input and a 3D filter applied. In comparison to 3D filters, we believe that utilizing a 2D CNN and applying two 1D filters, one along time and one along with space, will reduce computational complexity and improve the model’s ability to deal with subject-specific difficulty. According to researchers in [26], flattened networks, which use only one-dimensional filters to cover all three dimensions in 3D, perform as well as, or better than, conventional convolutional networks while using far less processing. The 3D filter is more difficult to implement in real-time applications than the 1D filter.

A multi-branch model’s fundamental concept is that the raw or prepared input is routed through multiple subnetworks, each with its own set of characteristics. The authors of [27] developed a CP-MixedNet architecture that used multiscale EEG features extracted from a series of convolution layers, each of which captures EEG temporal information at various scales. In [28] the authors propose a parallel spatial-temporal representation of raw EEG signals that makes use of the self-attention process to generate separate spatial-temporal features. To encode spatial correlations between MI EEG channels, they exploited the spatial self-attention module in particular. Additionally, the temporal self-attention module transforms global temporal information into sample time step characteristics, enabling time-domain extraction of high-level temporal aspects in MI EEG data. The authors of [29] divided the original signal into three band-limited signals by filtering it across separate band ranges. They varied the size of the temporal convolutional filter in each band range, resulting in nine parallel branches, three for each filter band. This resulted in a massive number of parameters totaling over 1215 K for the entire system and 405 K for a single filter band. As a result of this limitation, the system’s application in a wide variety of applications is limited. Furthermore, because the filter size did not change, the method did not account for the impact of shifting neighborhoods in channels.

The authors proposed a more advanced method in [30]. It is a temporal-spectral-based squeeze-and-excitation feature fusion network (TS-SEFFNet). In a cascade architecture, the deep-temporal convolution block (DT-Conv block) is the first section of their model, which employs convolutions to extract high-dimension temporal representations from raw EEG data. The multispectral convolution block (MS-Conv block) is then run in parallel using multilayer wavelet convolutions to capture discriminative spectral information from matching sub-bands. The final recommended block was the squeeze-and-excitation feature fusion block (SE-Feature-Fusion block), which was used to fuse deep-temporal and multispectral data into comprehensive fused feature maps. Interdependencies between different domain characteristics are introduced, bringing channel-specific feature responses to the forefront. It is a sizable model with numerous parameters (282 K).

In [31], a hybrid of the multi-scale and an attention mechanism was presented. The authors built a multi-scale fusion convolutional neural network based on the attention process (MS-AMF). To maintain as much information flowing as possible, the network captures spatiotemporal multi-scale characteristics from multi-brain area representation signals and applies a dense fusion mechanism. The network’s sensitivity was increased by the attention method they used, which consisted of Squeeze-and-Excitation (SE). However, before the data are entered into the model, this model includes a part for data preparation. Jia et al. [32] suggested an end-to-end approach for decoding raw EEG signals that do not include any pre-processing or filtering or Multibranch Multi-scale Convolutional Neural Network (MMCNN). It is a huge model with several branches at each scale, which increases its complexity and results in a high number of parameters. It is composed of five parallel branches that each contain an EEG Inception block, a residual block, and an SE.

Our suggested model, in contrast to existing multibranch, multiscale, and parallel networks, takes advantage of the essential element of multibranch with a kernel size fluctuation to improve classification accuracy while maintaining a low level of complexity and a limited number of parameters.

## 3. Materials and Methods

### 3.1. EEG Data

The three major components of a traditional MI EEG-based classification system are pre-processing, feature extraction, and classification. A preprocessing procedure is performed to reduce noise and artifacts from raw EEG data. It is not a requirement, although it is utilized in many systems. In this study, we do not perform any fundamental preprocessing on the raw data to make the model more applicable to real-world applications; rather, we extract the motor imagery time frame from the trail. There is no more bandpass filtering. On the other hand, feature extraction from EEG data is a critical step before classification because it identifies the motor movement imagined by the subject.

We want to validate the proposed model using multiple datasets with varied settings. The BCI Competition IV dataset 2a (BCI-IV2a) and the high Gamma dataset (HGD) were both used in this experiment. With 22 electrodes and a sampling frequency of 250 Hz, the BCI IV 2a was recorded from 9 subjects. We retrieved 0.5 s from the start of the pre-cue to the end of each trial, for a total trial duration of 4.5 s (250 × 4.5 = 1125 samples). There was no additional prepossessing for each channel. Each trial took the shape of a dimensioned matrix (22 × 1125). For the HGD dataset, which was recorded from 14 subjects, we downsampled the data from 500 Hz to 250 Hz. Furthermore, the number of channels was lowered from 128 to 44 to avoid unnecessary information. We excluded the electrodes not connected to the motor imagery area. We selected only sensors with ‘C’ (according to the dataset) in their name as they cover the motor cortex, which is 44 sensors. In addition, each trial has had a length of 4.5 s, resulting in (4.5 × 250) 1125 samples. The trial matrix had the following dimensions: (44 × 1125). There were no bandpass filters used, and each channel was standardized. It can be noted that the number of samples (trials) in the HGD is much more than in the BCI-IV2a dataset.

Because we wish to use a raw EEG signal without any preprocessing, we chose the full band for the dataset in this work. Here, full band means we are using all the frequency components from both datasets with a 250 Hz sampling frequency.

### 3.2. EEGNet Block

These three critical characteristics of the cerebral cortex that can be replicated using a CNN network are local connectivity, location invariance, and local transition invariance. CNNs, which articulate the convolution process within the context of a neural network [33,34], address the issue of high-dimensional input, such as EEG signals.

The EEGNet, developed in [19], serves as the building block for our proposed model. There are three types of convolution operations in the EEGNet block, each with different convolutional window sizes. The convolutional window, which is a small part of the input neurons, is connected to each neuron in the EEGNet’s hidden layer. A bias is assigned to each neuron, and a weight is assigned to each link. The window of the hidden layer is then scrolled across the entire input sequence, and each neuron learns to investigate a different part of it. The kernel size determines the size or length of the convolutional window. Rather than learning new weights and biases for each hidden layer neuron, the EEGNet now learns a single set of weights and biases for all hidden layer neurons. The weight-sharing principle is as follows:

(1) $$a_{ij}=f\left(b_{i}+\overset{k}{\underset{K=1}{\sum }}w_{iK}x_{j+K-1}\right)=f\left(b_{i}+W_{i}^{T}X_{j}\right)$$

where *a_ij_* is the activation or output of the *j*th neuron of the *i*th filter in the hidden layer, *f* corresponds to the activation function, *b_i_* is the shared overall bias of filter *i*, *K* is the kernel size, *W_i_* = [*w_i*1*_ w_i*2*_ … w_ik_*] is a vector of the shared weights and *X_j_* = [*x_j_ x_j+*1*_ … x_j+k−*1*_*] is a vector of the output of the previse neurons, and *T* denotes the transpose operation.

**Figure 1:**
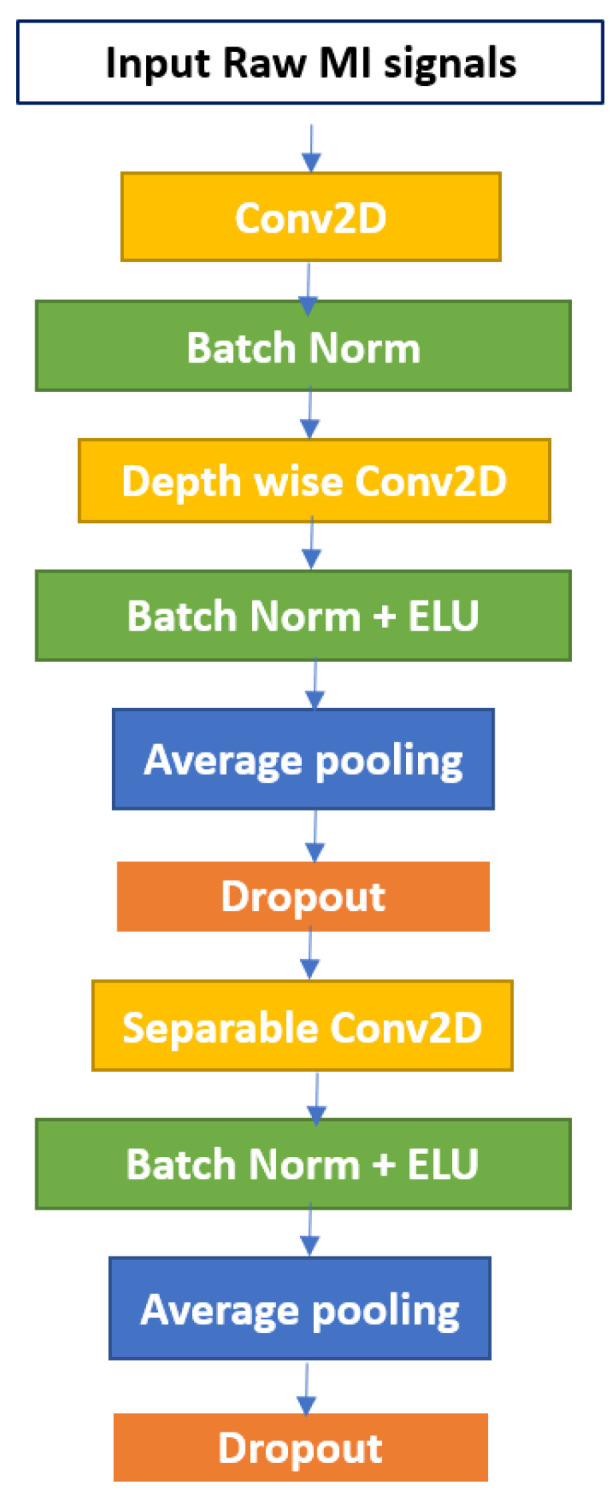
The EEGNet Block.

The EEGNet block first learns frequency filters via 2D temporal convolution, and then spatial filters via depth-wise convolution. Before combining and categorizing the feature maps, separable convolution learns a temporal summary for each. Batch normalization, pooling layers, and dropout are the remaining layers of EEGNet. Each of these layers has several tweakable parameters and performs different tasks on the input data. Batch normalization is a technique for normalizing the layers of a neural network rather than the raw input. Instead of using the entire dataset to normalize it, mini-batches are used. Batch normalization helps with training acceleration, and learning facilitation, enables the use of higher learning rates, and model regularization also helps to prevent overfitting [35]. The pooling layer, on the other hand, reduces the dimensionality of each map while preserving important data. Spatial pooling, also known as subsampling or down-sampling, takes a variety of forms. Max-pooling and average pooling are the two most well-known types. Additionally, the dropout probability is used to turn off some neurons to reduce the number of parameters. The composition structure of the EEGNet block is depicted in Figure 1.

### 3.3. SE Attention Block

One of the most fundamental properties of the human visual system is that it does not attempt to process an entire scene at once. To better capture visual structure, humans employ a succession of fragmentary glimpses and selective focus on critical areas of the image [36]. Deep learning’s attention mechanism is based on this concept. It is a block that can be used in conjunction with an existing model to improve performance by focusing on critical elements and suppressing non-critical ones.

**Figure 2:**
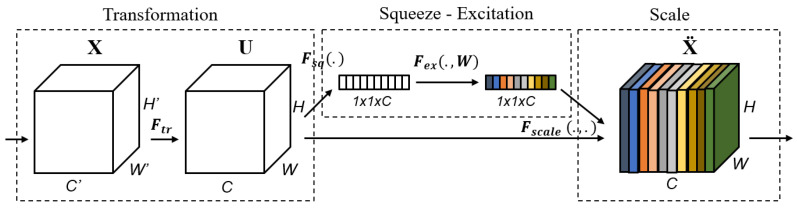
The Squeeze-and-Excitation (SE) Block.

The SE block is one of the attention blocks, as described in [37]. The authors assert that the convolutional output results in entangled channel dependencies due to the spatial correlation captured by the filters. This was accomplished by combining three primary components, as illustrated in Figure 2. It is critical to note that the middle section contains only the squeeze and excitation steps, whereas the first and last sections contain the transformation and scaling operations, respectively. By calibrating the extracted features, the SE block can increase the output volume of a transformation operation. It is a computational unit that begins with a transformation that converts an input *X* to feature maps *U* and then performs average pooling at each channel to construct a squeezed representation of the volume *U* in the squeezing step. Before the sigmoid-activated gating network, a new parameter called the reduction ratio *r* is used in the excitation stage to introduce a first fully connected (FC) layer with a ReLU activation. The objective is to create a bottleneck that enables us to decrease the dimension of the system while simultaneously introducing new non-linearities. Additionally, we can exert greater control over model complexity and improve the generalization property of the network. Scaling is the final phase, and it is a procedure for re-scaling. We will restore the squeezed vector to its original shape while retaining the information gathered during the excitation step. Scaling mathematically is accomplished by multiplying each channel on the input volume by the corresponding channel on the activated 1 × 1 squeezed vector.

### 3.4. Proposed Models

The ideal kernel size for motor imagery varies from subject to subject and from time to time for the same subject, according to the literature [4]. To overcome the subject-specific difficulty in EEG MI classification, we proposed an EEG MI multi-branch classification model, with each branch having its own set of parameters. The proposed method attempts to determine the optimal convolution size, filter count, dropout probability, and attention parameters for each individual. The technique can be subject-specific while also broadening the model’s scope through the use of appropriate parameters. The model is built to learn temporal properties from the first convolutional layer using temporal hierarchies of local and global modulations, as well as spatial features from the second convolutional layer using spatially global unmixing filters. The input data are represented as a two-dimensional array, with the number of electrodes represented by rows and the number of time steps represented by columns. The MI-EEG signal dataset is represented as follows:

(2) $$D={\left\{S_{i},\;L_{i}\right\}}_{i=1}^{t}$$

where *S_i_*, *L_i_* are the signal and their corresponding class labels, *t* is the number of trials, and *L_i_* ∈ {1, 2, …, *n*}, where *n* is the number of classes. *S* is represented as the input signal; it is a 2D array, *S* = [*C T*] where *C* refers to the number of EEG channels and *T* to the length of EEG signal input. The output of the final layer, which is a softmax layer with a softmax activation function, is the classification output. This layer produces a vector with the probability of each possible outcome or class. The sum of the probability in the vector for all conceivable outcomes or classes is one. The softmax can be defined as follows:

(3) $$F{\left(v\right)}_{i}=\frac{e^{v_{i}}}{\sum_{j=1}^{n}e^{v_{i}}}$$

where *v* is the input vector to the softmax function *F*; it contains *n* elements for *n* outcomes, *v_i_* is the *i*th element in the input vector *v*, and *n* is the number of classes.

The proposed method, MBEEGSE, is composed of two components: the EEGNet blocks and the SE Blocks. Both basic blocks have layers similar to those described in [19,37]. The EEGNet block learns frequency filters using a 2D temporal convolution, and then frequency-specific spatial filters using a depth-wise convolution, while the separable convolution learns a temporal summary for each feature map separately before mixing and classifying the feature maps. The SE is a straightforward gating mechanism in channel-based interactions. To simplify, when networks use the SE block, they can learn to recognize the importance of each feature map in a stack of all the feature maps extracted following a convolution operation and adjust the output to reflect that importance before transferring the volume to the next layer.

**Figure 3:**
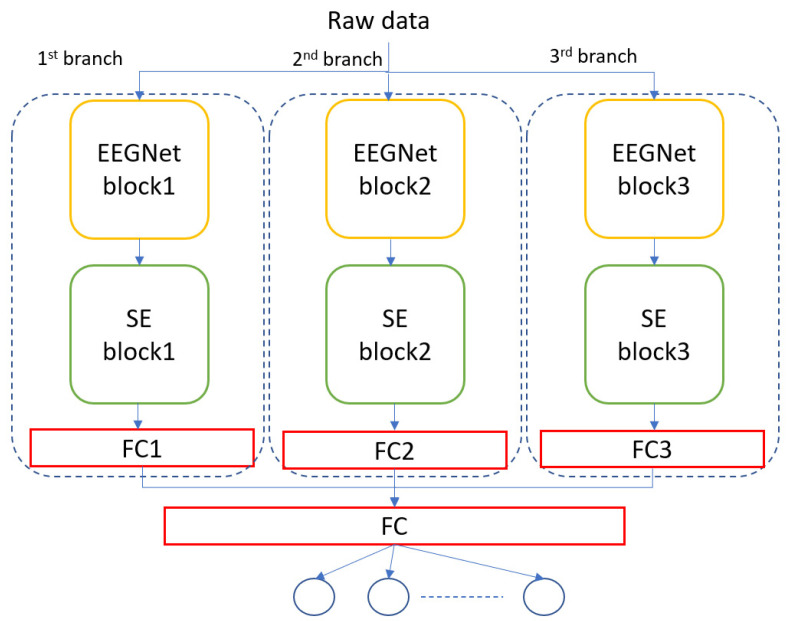
The architecture of the proposed model, MBEEGSE.

Figure 3 shows the architecture of the MBEEGSE. It is divided into three branches, each with an EEGNet and SE block as well as a fully connected layer. Concatenating the output of the three branches results in the addition of another fully connected layer, followed by a softmax layer for classification. Each branch has a different number of parameters to collect distinct features from all parts of the signal. Our model was evaluated using two benchmark datasets for MI EEG classification: the BCI-IV2a and the HGD.

## 4. Results and Discussion

The mental and physical states of research subjects can vary substantially in EEG-MI studies. To accomplish this, we classified the data in this study using the within-subject technique. To put it another way, the model is trained and tested using data from multiple sessions recorded for the same person [22]. The proposed model is employed in this study to apply the within-subject technique to both the BCI-IV2a and the HGD datasets. One session is utilized for training and the other is used for testing both datasets. Global parameters are used for all individuals in the proposed model for both datasets, as indicated in Table 1. We previously examined the optimal hyperparameters for the EEGNet blocks in [38]. During the training phase, a callback is used to save the best model weights based on the current best accuracy, and the best-saved model is then loaded during the test phase. With a batch size of 64 and a learning rate of 0.0009, the model is trained for 1000 epochs. For the cost function, a cross-entropy error function was constructed and an Adam optimizer was used. All experiments were done in Google’s Colab environment making use of the Tensorflow deep learning library and the Keras API.

### 4.1. Overall Comparison

Using the aforementioned BCI-IV2a and HGD datasets, the performance of the recommended strategy is compared to that of open-source end-to-end models and alternative multibranch methods.

1. **FBCSP** is a handcrafted model for classifying motor imagery EEG data that are often used as a baseline method [17]. It won several EEG decoding competitions, including the BCI competition IV in both datasets 2a and 2b. The CSP features are retrieved from different frequency bands in this model before being classified using the SVM [17].
2. **ShallowConvNet** is a deep learning network that can categorize MI-EEG with only two convolution layers and a mean pooling layer [11].
3. **DeepConvNet** is a deeper deep learning model than ShallowConvNet. It consists of four convolution and max-pooling layer blocks, followed by a softmax layer [11].
4. **EEGNet** is a deep learning model that uses two-dimensional temporal convolution, depthwise convolution, and separable convolution to achieve a consistent approach to various BCI tasks [19].
5. **CP-MixedNet** is a multi-scale model that extracts EEG features from many convolution layers, each of which captures EEG temporal information at different scales [27].
6. **TS-SEFFNet** is a multi-block system that employs attention and fusion techniques. The spatio-temporal block, the deep-temporal convolution block, the multi-spectral convolution block, the squeeze-and-excitation feature fusion block, and the classification block are all part of a larger model [30].
7. **CNN + BiLSTM** (fixed) is a hybrid deep learning model which contains an attention-based inception model and the LSTM model. It was tested and analyzed with fixed hyperparameter values, which were fixed for all subjects [15].

We also compared our findings to earlier research [38], which included lightweight multibranch models without attention blocks, Multi-branch EEGNet (MBEEGNet), and Multi-branch ShallowConvNet (MBShallowConvNet). As seen in Table 2, the attention block improves accuracy by about 1%. Table 2 summarizes the classification accuracies achieved from the BCI-IV2a and HGD datasets using the baseline models we mentioned above. As can be shown, our approaches have the highest average accuracy, kappa, and F1 score. It can be noted that we compared our result with results achieved by the same training method (the within-subject).

### 4.2. Results of BCI Competition IV-2a Dataset

All of the proposed models were trained using session “T” from the BCI-IV2a data set and tested on session “E.” In the experiments, a subject-specific method was used. Classification accuracy, Cohen’s score, precision, recall, F1 score, and the number of parameters were all employed to compare the proposed model against state-of-the-art MI-EEG classification models.

**Figure 4:**
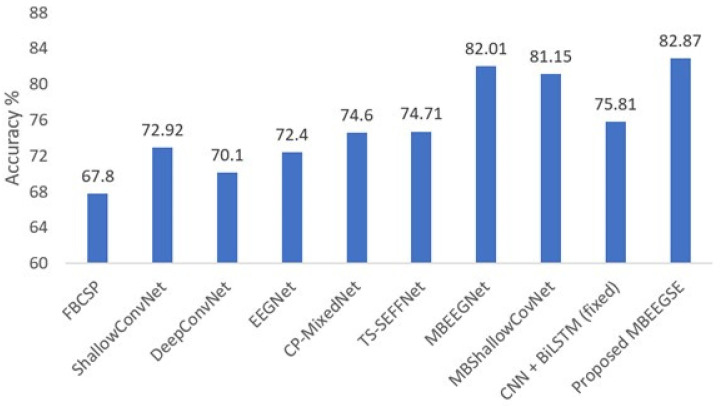
Average classification accuracy on the BCI-IV2a dataset.

Figure 4 illustrates our method’s performance in comparison to the baseline models in BCI-IV2a. As shown in the figure, the proposed model outperforms other baseline models in the BCI-IV2a by more than 7% and at least 1% for the same model without attention blocks.

**Figure 5:**
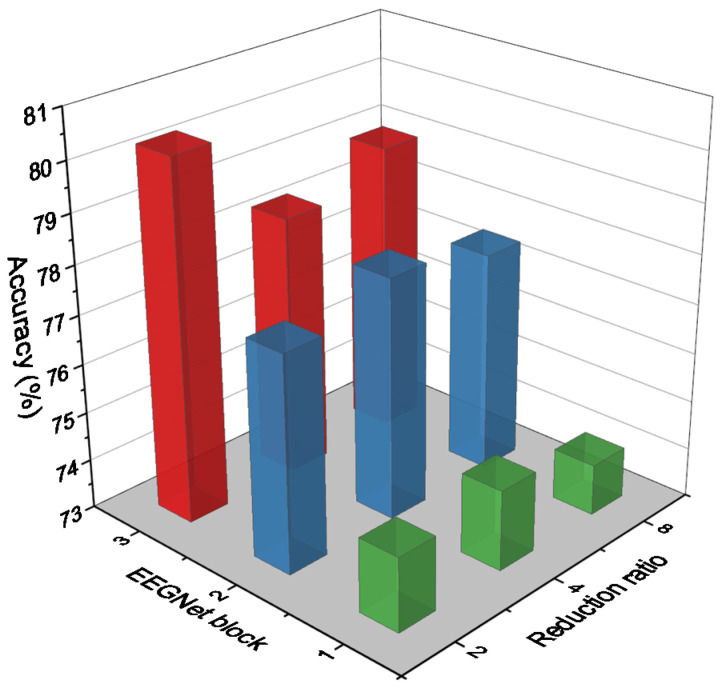
Accuracy comparison on different EEGNet blocks with different reduction ratios in SE block.

One of the study’s primary objectives is to identify the best hyperparameters in each branch that can improve classification accuracy with the least amount of complication. As a result, we begin by performing multiple experiments to determine the optimal hyperparameters in the EEGNet block [38]. Then, we conduct additional experiments to determine the optimal reduction ratio for the SE block. Figure 5 compares the accuracy of different redaction ratios in the SE block on various EEGNet blocks. As illustrated in Figure 5, EEGNet Block 3 with a different reduction ratio in the SE block outperforms other blocks by an average accuracy of around 79%. In EEGNet Block 1, the highest accuracy was obtained with a reduction ratio of 4. Reduction ratio 4 is more accurate in EEGNet Blocks 1 and 2, but ratio 2 is more accurate in EEGNet Block 3. The experiments revealed that the number of parameters increases with the number and size of filters in EEGNet Block and with the reduction ratio in SE Block. As a result, we selected a reduction ratio of 2 for EEGNet Block 3 and a reduction ratio of 4 for EEGNet Block 1 and Block 2. That was the set of hyperparameters we used in each branch of our proposed model in both datasets for the SE blocks as we mentioned in Table 1.

The proposed model was compared to state-of-the-art MI-EEG classification models using classification accuracy, Cohen’s score, precision, recall, and F1 score. Table 3 summarizes the findings from the BCI-IV2a dataset using MBEEGSE. Additionally, even with this increase in average accuracy, we were still working with a limited number of parameters. To gain a better understanding of the proposed method’s computational complexity, we calculate the number of parameters in our model and compare it to existing multi-branch techniques. As shown in Table 4, the proposed MBEEGSE has a total of 10,170 parameters across all branches, which is less than other multi-branch models such as TS-SEFFNet and CP-MixedNet, which have 282,000 and 836,000 parameters, respectively.

The time required to predict a motor imagery class from an EEG test sample was calculated using Python commands. According to the Google Colab environment’s specifications, our proposed model takes an average of 1.79 milliseconds to predict the class. Additionally, we calculate the information transfer rate (ITR), which is a critical evaluation metric when developing an embedded system. It is a widely used technique for assessing the communication performance of control systems, more specifically BCI [39,40]. The quantity of data transmitted per unit of time is referred to as the ITR. Typically, the ITR is expressed in bits/min using the following formula:

(4) $$ITR=T\left(log_{2}C+A\;log_{2}A+\left(1-A\right)\;log_{2}\frac{1-A}{C-1}\;\right)$$

where *T* is the number of decisions per minute, *C* stands for number of classes (in our case, we have four MI classes), and *A* for accuracy. As mentioned above, 4.5 s were used from each trial, so in a minute 13.33 trials can be processed. The average accuracy of the method is *A* = 0.8287 and the *ITR* achieved for each subject in the BCI-IV2a dataset is presented in Table 5. From the table, we can see that the average *ITR* achieved was 14.93 bit/min, which is a good value in BCI applications [41].

**Figure 6:**
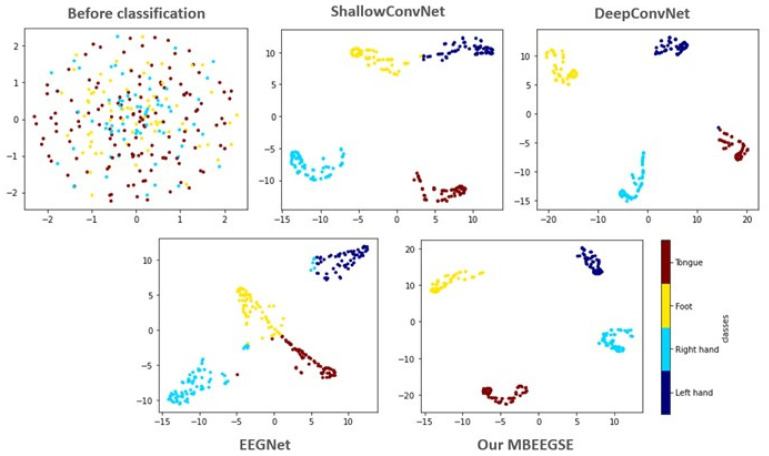
The t-SNE visualization in 2D embedding space of test sample before and after classified by different methods from the third subject in the BCI-IV2a.

To investigate the discrimination of the features extracted by our MBEEGSE in greater detail, the t-SNE is used to visualize the learned features. The t-SNE transforms the extracted EEG features into a two-dimensional embedding dimension, as illustrated in Figure 6. In comparison to ShallowConvNet [11], DeepConvNet [11], and EEGNet [19], our MBEEGSE model implements multi-branch feature extraction and captures more MI-EEG features with fewer parameters. Additionally, the proposed model’s feature visualizations demonstrated that it was capable of extracting both temporal and spectral features from EEG signals. Additionally, the proposed MBEEGSE generates more separable features than the EEGNet, enabling it to distinguish between different types of MI-EEG signals efficiently. As a result, we can see that our MBEEGSE extracts the most discriminative EEG features, implying the highest decoding performance.

### 4.3. Results of HGD

**Figure 7:**
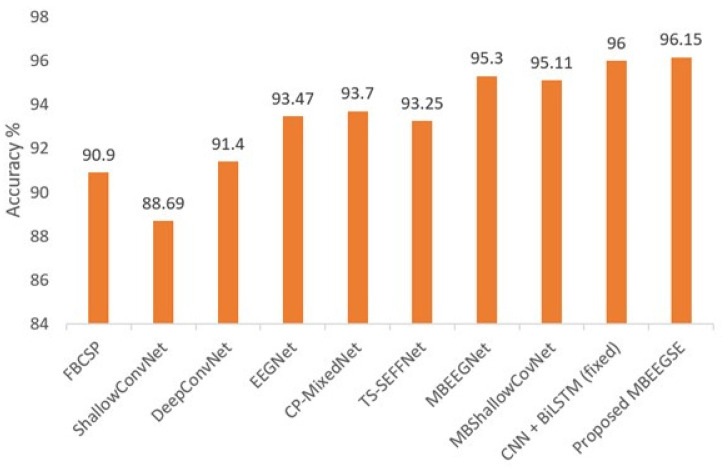
Average classification accuracy on the HGD.

The accuracy, kappa value, precision, recall, and F1 scores for each subject in the second dataset (HGD) are summarized in Table 6. Moreover, in the same dataset, the average classification accuracies of our proposed multibranch model (MBEEGSE) are shown in Figure 7 in comparison to the single-scale models FBCSP [17], ShallowConvNet [30], DeepConvNet [11], EEGNet [38], and other multiscale networks CP-MixedNet [27], TS-SEFFNet [30], and CNN + BiLSTM (fixed) [15]. The findings indicate that our model effectively addresses the issue of subject and session (time) difference, thereby increasing the accuracy of MI classification.

## 5. Conclusions

We proposed MBEEGSE, which is a lightweight multibranch model with attention blocks capable of increasing EEG MI classification accuracy while utilizing fewer parameters. Two publicly available datasets, BCI-IV 2a and HGD, were used to validate the performance of the model. The average accuracy and F1 score of the proposed model were 82.87% and 0.829 using the BCI-IV 2a dataset, and 96.15% and 0.962 using the HGD, respectively. The proposed model outperformed the base EEGNet model by more than 10% accuracy, and the multibranch EEGNet without attention blocks by 0.86% accuracy when using the within-subject strategy in the BCI-IV 2a dataset. Similarly, the proposed model performed better than other compared models using the HGD. Two major findings of this study are as follows:

- The self-attention mechanism increases the accuracy of EEG-MI classification.
- By applying variable optimum reduction ratios of the attention mechanism in different branches, we can reduce the number of hyperparameters in the multibranch model of the EEG-MI classification.

Compared to the base EEGNet, the proposed model has 3.9 times more the number of hyperparameters; however, the accuracy was improved by more than 10%. Though the number of hyperparameters is larger than that in the EEGNet, we can utilize the parallel processing of three branches as they are independent of each other in the proposed model. This will significantly reduce the processing time.

In the future, we intend to investigate various attention strategies to increase the accuracy of EEG-MI classification models and develop models that can be used in advanced BCI systems. Another direction of the future work can be to investigate on which frequencies the model should give more attention for a better accuracy than the proposed model.

## Figures and Tables

**Table 1:** Global hyper-parameters used in proposed model.

Branch	Block	Activation Function	Hyperparameter	Value
First branch	EEGNet Block	ELU	Number of temporal filters	4
			Kernel size	16
			Dropout rate	0
	SE Block	ReLU	Reduction ratio	4
Second branch	EEGNet Block	ELU	Number of temporal filters	8
			Kernel size	32
			Dropout rate	0.1
	SE Block	ReLU	Reduction ratio	4
Third branch	EEGNet Block	ELU	Number of temporal filters	16
			Kernel size	64
			Dropout rate	0.2
	SE Block	ReLU	Reduction ratio	2

**Table 2:** The comparison summary of classification performance in proposed models.

Datasets	Methods	Accuracy (%)	Kappa	F1 Score
BCI-IV2a	FBCSP [17]	67.80	NA *	0.675
	ShallowConvNet [29]	72.92	0.639	0.728
	DeepConvNet [11]	70.10	NA	0.706
	EEGNet [20]	72.40	0.630	NA
	CP-MixedNet [26]	74.60	NA	0.743
	TS-SEFFNet [29]	74.71	0.663	0.757
	MBEEGNet [37]	82.01	0.760	0.822
	MBShallowCovNet [37]	81.15	0.749	0.814
	CNN + BiLSTM (fixed) [15]	75.81	NA	NA
	Proposed (MBEEGSE)	82.87	0.772	0.829
HGD	FBCSP [17]	90.90	NA	0.914
	ShallowConvNet [29]	88.69	0.849	0.887
	DeepConvNet [11]	91.40	NA	0.925
	EEGNet [37]	93.47	0.921	0.935
	CP-MixedNet [26]	93.70	NA	0.937
	TS-SEFFNet [29]	93.25	0.910	0.901
	MBEEGNet [37]	95.30	0.937	0.954
	MBShallowCovNet [37]	95.11	0.935	0.951
	CNN + BiLSTM (fixed) [15]	96.00	NA	NA
	Proposed (MBEEGSE)	96.15	0.949	0.962

* NA means Not Available.

**Table 3:** Performance Metrics on the BCI-IV 2a dataset using the MBEEGSE.

		1	2	3	4	5	6	7	8	9	Avg.	Std. Dev.
Accuracy (%)		89.14	69.73	95.27	81.42	80	63.25	94.06	89.57	83.35	82.87	0.108
K value		0.855	0.596	0.937	0.752	0.733	0.510	0.921	0.861	0.778	0.772	0.144
F1 score		0.892	0.696	0.953	0.816	0.800	0.633	0.943	0.896	0.835	0.829	0.108
Precision	LH	0.857	0.602	0.955	0.872	0.760	0.594	0.967	0.968	0.857	0.826	0.145
	RH	0.926	0.563	0.932	0.760	0.917	0.660	0.905	0.915	0.769	0.816	0.136
	F	0.906	0.850	0.954	0.718	0.739	0.703	0.934	0.857	0.871	0.837	0.094
	Tou.	0.876	0.774	0.970	0.907	0.783	0.574	0.956	0.843	0.837	0.836	0.120
	Avg.	0.891	0.697	0.953	0.814	0.800	0.633	0.941	0.896	0.834	0.829	0.108
Recall	LH	0.907	0.690	0.958	0.824	0.833	0.626	0.846	0.907	0.833	0.825	0.106
	RH	0.910	0.586	0.984	0.750	0.868	0.611	0.965	0.939	0.785	0.822	0.149
	F	0.859	0.832	0.917	0.896	0.774	0.636	0.984	0.869	0.797	0.840	0.099
	Tou.	0.892	0.675	0.955	0.805	0.728	0.661	0.987	0.868	0.931	0.833	0.122
	Avg.	0.892	0.696	0.953	0.819	0.801	0.634	0.945	0.896	0.837	0.830	0.109

Where LH: Left Hand, RH: Right Hand, F: Feet, Tou.: Tongue.

**Table 4:** Comparison of the number of parameters and mean accuracy using BCI-IV2a dataset.

Methods	Mean Accuracy (%)	Number of Parameters
FBCSB [38]	73.70	261 × 10^3^
ShallowConvNet [20]	74.31	47.31 × 10^3^
DeepConvNet [29]	71.99	284 × 10^3^
EEGNet [20]	72.40	2.63 × 10^3^
CP-MixedNet [29]	74.60	836 × 10^3^
TS-SEFFNet [29]	74.71	282 × 10^3^
MBEEGNet [37]	82.01	8.908 × 10^3^
MBShallowConvNet [37]	81.15	147.22 × 10^3^
CNN + BiLSTM (fixed) [15]	75.81	55 × 10^3^
Proposed (MBEEGSE)	82.87	10.17 × 10^3^

**Table 5:** ITR values for each subject in the BCI-IV2a dataset.

Subject	ITR (Bits/Min)
S1	17.76
S2	8.47
S3	22
S4	13.50
S5	12.81
S6	6.25
S7	21.07
S8	18.02
S9	14.48
Average	14.93

**Table 6:** Performance metrics on the HGD dataset using the MBEEGSE.

Subject/Metric	Accuracy (%)	K Value	Precision	Recall	F1 Score
S1	97.05	0.961	0.971	0.971	0.971
S2	95.14	0.935	0.952	0.953	0.952
S3	100	1	1	1	1
S4	98.80	0.984	0.988	0.988	0.988
S5	98.15	0.975	0.981	0.982	0.982
S6	99.40	0.992	0.994	0.994	0.994
S7	93.84	0.918	0.938	0.939	0.939
S8	96.75	0.957	0.968	0.971	0.969
S9	98.77	0.984	0.988	0.988	0.988
S10	92.77	0.904	0.928	0.930	0.929
S11	94.70	0.929	0.947	0.948	0.948
S12	97.49	0.967	0.975	0.975	0.975
S13	96.25	0.950	0.963	0.963	0.963
S14	87.02	0.827	0.870	0.874	0.872
Average	96.15	0.949	0.962	0.963	0.962
Std. Dev.	0.034	0.045	0.034	0.033	0.033

## Data Availability

The BCI-IV2a dataset can be downloaded from the following link: http://www.bbci.de/competition/iv/#dataset2a (accessed on 30 December 2021), and the HGD dataset can be downloaded from the following link: https://gin.g-node.org/robintibor/high-gamma-dataset (accessed on 30 December 2021).
